# Potential Markers to Reduce Non-Contrast Computed Tomography Use for Symptomatic Patients with Suspected Ureterolithiasis

**DOI:** 10.3390/jpm12081350

**Published:** 2022-08-21

**Authors:** Yuval Avda, Igal Shpunt, Jonathan Modai, Dan Leibovici, Brian Berkowitz, Yaniv Shilo

**Affiliations:** 1 Department of Urology, Kaplan Medical Center, Affiliated with the Hebrew University, Rehovot 7661041, Israel; 2 Department of Earth and Planetary Sciences, Weizmann Institute of Science, Rehovot 7610001, Israel

**Keywords:** urolithiasis, renal colic, clinical score, imaging

## Abstract

Most patients with ureterolithiasis are managed successfully with conservative treatment. In this context, delineation of clinical risk factors that identify patients with low risk for surgical intervention may reduce use of Non-Contrast Computed Tomography (NCCT). Here, emergency department patient files from a 14-month period were reviewed retrospectively, to identify patients who underwent NCCT and showed a ureteral stone. Demographic, clinical and laboratory information was collected. Patients were grouped to either requiring surgical intervention (Group 1) or having successful conservative management (Group 2). The cohort included 368 patients; 36.1% ultimately required surgical intervention (Group 1) and 63.9% were successfully treated conservatively (Group 2). On univariate analysis, patients who required surgical intervention were older, had longer duration of symptoms, had history of urolithiasis and surgical intervention for urolithiasis and had higher serum creatinine levels. Multivariate analysis identified the following risk factors associated with surgical intervention: creatinine >1.5 mg/dL, duration of symptoms ≥ 1.5 days and age > 45 years. Patients with 0, 1, 2 or 3 of the identified risk factors had 19%, 32%, 53% and 73% likelihood, respectively, of surgical intervention. Incorporating these data may reduce the use of NCCT scans in patients who are likely to pass a stone via conservative management.

## 1. Introduction

Renal colic is a common and increasing symptom responsible for emergency department (ED) visits, with estimates of more than 1 million annual occurrences in the United States [1,2]. The increase in ED visits is reported, specifically, in young and female patients [3].

Concurrently, use of imaging as a diagnostic tool in patients presenting with renal colic is also rising, with NCCT being the most commonly used imaging tool under such circumstances [1,4]. This is due, evidently, to the high sensitivity and specificity of NCCT for the detection of ureterolithiasis. However, only a minority of patients presenting with renal colic actually requires admission and/or surgical intervention [5]—even for known ureterolithiasis, approximately 65–90% of cases resolve without surgical intervention, depending mostly on stone size and location [5,6,7,8]. Therefore, it is questionable whether broad use of NCCT improves outcomes [1]. Moreover, radiation exposure is not negligible, with reported median exposure as high as 30 mSv in the first year following the diagnosis of urolithiasis, and 17–20% of patients with exposure levels exceeding 50 mSv [9,10], although recent reported exposure levels are generally lower (7.0–26 mSv) [11,12,13]. These radiation levels are associated with increased risk of cancer, especially in young female patients [12,14,15,16,17]. Accordingly, there is a pressing need to determine clinical approaches for improved assessment of renal colic patients, in an effort to reduce radiation exposure from the use of NCCT.

When considering efforts to decrease the demand use of NCCT for patients with renal colic, several advantages of NCCT scans should be recognized: (i) a high level of accurate diagnosis regarding stone presence, (ii) a high level of certainty that an important alternative diagnosis has not been recognized, and (iii) confidence to advise patients on conservative management versus surgical intervention. This last point is based mostly on determination of stone size and location, with small distal stones being more likely to be expelled spontaneously, and large proximal stones more likely to be treated surgically [5,18]. This approach is supported by American Urological Association guidelines [19]. One must recognize, however, that identification of patients with small distal stones by NCCT does not guarantee successful conservative treatment. This failure rate should be compared to any failure rate of alternative methods of identifying patients suitable for conservative treatment.

We hypothesize that the need for surgical intervention in renal colic ED patients—who are ultimately diagnosed with ureterolithiasis—can be predicted clinically, with a significant likelihood of success, without the need for a priori (general) use of NCCT. Identification of patients with low risk for surgical intervention could decrease the use of NCCT and radiation exposure in these patients, while saving NCCT scans for patients with higher risk for intervention where it is certainly required.

## 2. Materials and Methods

### 2.1. Patient File Selection

The institutional review board approved this study. We retrospectively reviewed the institutional imaging software (PACS, version 11, Minnetonka, MN, USA) for all NCCT scans performed in the ED between March 2016 and May 2017. Inclusion criteria included detection of a single ureteral stone by NCCT, body temperature ≤ 37.8 °C, and sufficient background data as described below. Our intention was to differentiate between patients who will require surgical intervention and those who can be managed with a conservative approach. The combination of ureterolithiasis and fever generally requires urgent drainage and we therefore excluded these patients. We intentionally selected patients with known, single ureteral stones identified by NCCT, focusing on identification of risk factors specific to those patients that ultimately required surgical intervention.

From these patient files, we collected demographic, clinical and laboratory data that were available to the treating ED physician on all such patients. This included age, gender, duration of symptoms, pain level at presentation according to Visual Analog Scale score, nausea and/or vomiting, history of urolithiasis and associated intervention, and laboratory tests including blood count and creatinine level. Urinalysis is routinely assessed for all patients in the ED, but precise data on many patients with microhematuria were lacking; in any case, this marker is considered weak and less sensitive for ureterolithiasis [20]. Moreover, while microhematuria may be useful for diagnosis of ureterolithiasis, its role in prediction of surgical intervention for patients with ureteral stones appears generally less relevant.

The cohort was divided into patients who required surgical intervention (Group 1), and patients who were successfully managed conservatively (Group 2). We considered conservative management to be successful for patients who fulfilled all of the following: (1) did not require any surgical intervention, (2) no symptoms for at least two months (recognizing the definition for successful conservative management for ureterolithiasis in previous studies, which stipulated at least one month free of symptoms and with no need for surgical intervention [7]), (3) lack of evidence of hydronephrosis or stone on follow-up imaging, and (4) normal creatinine level. This practical endpoint has been used successfully elsewhere, and negates the use of repeat NCCT in many patients [7]. For Group 1 patients, surgical interventions included either drainage of the affected renal unit or primary ureteroscopy. The decision whether or not to intervene was left to the discretion of the attending urologist according to commonly accepted indications, such as intractable pain, elevated creatinine levels, large and proximal ureteral stones, presence of peri-renal urinoma and recurrent ED visits.

Subsequently, for the Group 1 patients, we obtained data on intervention for the ureteral stone using a unique, integrated hospital-community electronic medical record database (OFEK). This system provides data on the patient’s family doctor visits, hospital outpatient visits, laboratory and imaging results, both at the medical center, and in the community. Visits to other medical centers are also documented. Depending on the health care provider, the completeness of data varied. Therefore, when data on surgical intervention were unclear, we contacted the patient by phone. Cases were excluded if the outcome could not be determined clearly from the medical file, and the patient could not be contacted.

### 2.2. Statistical Analysis

The continuous variables were tested for normality using the Kolmogorov-Smirnov test. We compared a range of variables between groups using a *t*-test to compare the groups for age, a Mann-Whitney test to compare the other continuous variables, and a chi-square test for categorical variables. We selected cut-off points for continuous variables after plotting receiver operating characteristic (ROC) curves. Following the univariate analysis, we performed a multivariate logistic regression analysis that included all variables found to be statistically significant in the univariate analysis. Variables found to be statistically significant on the logistic regression were included in a score, with each variable being weighted equally and contributing 1 point. Patients with missing data of any of the risk factors were not scored, and were excluded from the “score cohort”. Patients with zero risk factors were defined as the “clinical low risk”. As a benchmark for successful conservative management, we defined the standard index patient suitable for conservative management following NCCT as any patient with small (≤5 mm) distal ureterolithiasis. This group was defined as the “NCCT low risk”. No formal comparative statistical tests were planned between these sub-groups. In all statistical analyses, *p* < 0.05 was considered statistically significant. Calculation of all statistical tests was performed using IBM SPSS Statistics version 21, Armonk, NY, USA, 2020.

## 3. Results

The cohort meeting the inclusion and exclusion criteria included 368 patients. Baseline characteristics are presented in Table 1. The average age was 47.2 years and the average duration of symptoms prior to ED arrival was 4 days. All patients had a ureteral stone found on the NCCT scan, and the average stone diameter was 5 mm.

Of the 368 patients, 110 of the stones were proximal to the iliac vessels, and 258 were distal. Hydronephrosis was present in 92% of the cases. Admission rate of this cohort was 48%, and readmission rate following a previous ED visit was 13%. Group 1 included 133 patients (36.1%) who ultimately required surgical intervention for their ureteral stone, and Group 2 included 235 patients (63.9%) who were successfully treated conservatively. Within Group 1, median time from ED visit to surgical intervention was 2 days.

Univariate analysis (Table 2) found that patients who required surgical intervention were older (50 vs. 46 years; *p* = 0.003), had longer duration of symptoms prior to ED arrival (6.5 vs. 2.7 days; *p* < 0.002), were more likely to have a past history of urolithiasis (53% vs. 36%; *p* = 0.002) and of surgical intervention for urolithiasis (13% vs. 6%; *p* = 0.033) and were found to have higher serum creatinine levels (1.32 vs. 1.14; *p* = 0.001).

**Figure 1:**
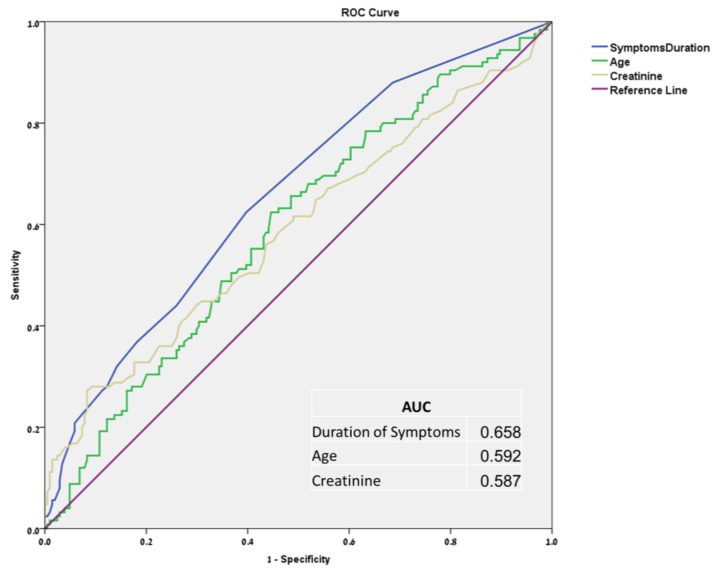
Receiver operating characteristic curves and area under the curve for symptoms duration, age, and serum creatinine level.

Using ROC curves (Figure 1), cut-off levels were defined for serum creatinine (1.5 mg/dL), duration of symptoms (1.5 days) and age (45 years). Using these cut-off levels, multivariate analysis (Table 3) further narrowed the statistically significant risk factors, identifying three key criteria as predictors for surgical intervention: serum creatinine level > 1.5 mg/dL (OR 2.8, 95% confidence interval (CI) [1.5–5.6]), duration of symptoms prior to ED arrival > 1.5 days (Odds Ratio, OR 2.3; 95% CI [1.4–3.8]) and age > 45 years (OR 1.7; 95% CI [1.02–2.7])). All three of these predictors of surgical intervention were correlated significantly to stone location and size, both individually and combined (Table 4).

**Figure 2:**
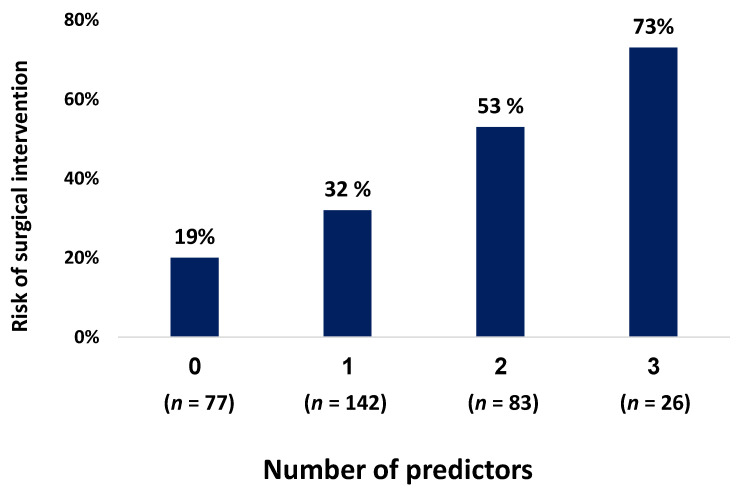
Risk of surgical intervention according to number of predictors.

We further stratified the entire cohort according to the number of risk factors for surgical intervention (Figure 2). Creatinine level and age were on file for all patients; 40 patients had symptom duration missing so they were excluded from the score analysis, leaving 328 patients in the “score cohort”. Patients with 0, 1, 2 or 3 of the identified risk factors had 19%, 32%, 53% and 73% likelihood, respectively, of surgical intervention (*p* < 0.0005). In particular, the “clinical low risk” group with none of the risk factors included 77 patients of the 328 “score cohort” (23.5%) and 81% of these patients—62 out of 77—ultimately had successful conservative management.

Additional analysis of the “NCCT low risk” group, included 189 patients with small distal stones. Of these patients, 164 did not require surgical intervention, leaving an 87% rate of successful conservative management in this group.

To summarize, the results indicate that higher creatinine level, longer duration of symptoms, and older age are risk factors for intervention in patients presenting to the ED with renal colic and ureterolithiasis. Additionally, all of these risk factors, individually and combined, are correlated to the higher likelihood of large and proximal stones on NCCT, as noted in Table 4.

## 4. Discussion

Our study attempted to define clinical risk factors for surgical intervention in renal colic patients with ureterolithiasis, with the specific aim of identifying clinical risk factors that might be used to avoid NCCT scans and associated risk of radiation exposure.

NCCT is used commonly in patients who present with renal colic at the ED, and frequently to identify patients with ureteral stone characteristics that carry low risk for intervention. Offering conservative management to such patients with small distal stones is probably the best initial management [6]. Our results suggest that by considering simple clinical and laboratory factors, nearly 25% of patients can be identified with low risk for intervention. Ultimately, 19% of the patients within this low-risk group will require surgery, while the remaining 81% will be managed successfully with a conservative approach. The clinical meaning is that most patients who satisfy these low-risk criteria will indeed be successful with a conservative approach, which justifies omitting the use of NCCT. This 19% failure rate is not notably different from the 13% rate of patients with small distal stones who ultimately underwent surgical intervention in our cohort. Note that a similar approach to minimize the use of NCCT for kidney stones exists in the treatment of nephrolithiasis in the pediatric population [21].

To minimize the use of NCCT, we must also be able to confidently advise patients that the diagnosis of ureterolithiasis is accurate, and that an important alternative diagnosis has not been missed. Our study did not approach these questions, which have been studied previously. In this context, for example, Moore et al. [22] suggested the STONE score, which was also validated by others [23]. This score predicts the presence of ureterolithiasis using five clinical factors: gender, duration of symptoms, race, nausea and vomiting, and microscopic hematuria, with the rate of ureterolithiasis in the high-risk group approaching 90%. The authors also suggested that the presence of hydronephrosis on ultrasound (US) be added to their score. Sternberg et al. [24] found that hydronephrosis on US has a positive and negative predictive value of 0.77 and 0.71 for the detection of ureterolithiasis, respectively. These studies suggest that patients can be diagnosed with ureterolithiasis based on clinical factors and US, with an acceptable level of confidence. In related studies, a large multi-center randomized controlled trial [25] found that US compared to NCCT reduces the 6-month cumulative radiation exposure, while maintaining a low and comparable rate of high-risk diagnoses with complications: 0.3% in the US group compared to 0.2% in the NCCT group (*p* = 0.3). Schoenfeld et al. [26] found no emergent or urgent findings in 115 patients 18–50 years old undergoing NCCT, concluding that the risk for dangerous alternative diagnoses or the need for emergent intervention in this patient group is very low (95% CI [0–2.7%]). These studies suggest that patients with flank pain have a low rate of serious alternative diagnosis, and US is safe compared to NCCT.

Taken together, these studies suggest that one can reasonably manage renal colic patients in the ED with the use of clinical, laboratory and US assessment, while maintaining high confidence in the diagnosis of ureterolithiasis, low rate of complications and low risk of failure of conservative management. A comprehensive approach based on such methods remains to be defined in future trials.

Notwithstanding the above, it is recognized that NCCT findings can pinpoint patients with very low risk for intervention. For example, we found that patients with distal ureteral stones with stone size < 4 mm, stone distance from the ureterovesical junction < 4 mm, and < 4 days of symptoms carry only 4.3% risk for surgical intervention [8].

The three risk factors identified here—creatinine level, duration of symptoms, and age—were found to correlate to stone size and location, contributing at least partly to their ability to predict surgical intervention. While it is understandable how higher creatinine and longer duration of symptoms can lead to a higher likelihood of surgical intervention, older age is less clear. A possible mechanism is that older age may be associated with less elasticity of the ureter and less tolerance to pain, compared to younger patients, but this hypothesis remains to be investigated further.

Previous studies have also evaluated risk factors associated with surgical intervention in patients with renal colic. In a prospective study including 220 patients, Yan et al. [27] found eight risk factors associated with risk for intervention. Their results found that being over 50 years old is a risk factor for surgical intervention, concurring with our observation. It is important to note that this study included CT information, and indeed stone size > 5 mm, and proximal location are two of the risk factors found. Wang et al. [28] performed a retrospective analysis of the results of a large multi-center randomized controlled trial, and found that in patients with renal colic, hydronephrosis, prior history of stone and WBC count can predict ureterolithiasis that will require surgical intervention within 30 days. This analysis did not include creatinine level because it was not captured in the original study. Our results did not find predictive value for WBC count.

Our analysis has several limitations. Given the retrospective nature of the study, only patients with NCCT-diagnosed, single ureteral stones were included, rather than all patients with renal colic. We therefore suggest that our findings here—indicating that higher creatinine level, longer duration of symptoms, and older age are the dominant risk factors for intervention in patients with known ureterolithiasis—should be validated prospectively for all patients presenting with renal colic. In addition, validation in other medical centers is important as clinical judgment for ordering a NCCT scan and the decision to intervene surgically may be influenced by local policies. Moreover, our study did not include US parameters, which may potentially increase the certainty of diagnosis of ureterolithiasis and assist in identifying patients with low risk for intervention [29]. Only a minority of patients in our cohort underwent US and we thus could not assess this important diagnostic tool; US offers an important benefit in the effort to minimize the use of NCCT for patients with renal colic. Future studies evaluating the combination of clinical parameters and US findings to predict surgical intervention are thus warranted.

## 5. Conclusions

It is an obligation to minimize radiation exposure, where possible, in patients presenting with renal colic, especially for patients with a history of ureterolithiasis. Because most patients with ureterolithiasis will eventually pass their stone spontaneously, defining clinical risk factors to minimize NCCT scan use appears attractive. Our results suggest that patients with low risk for intervention can be identified reasonably successfully on the basis of clinical and laboratory data, without NCCT. This finding, of course, is not intended to negate the importance of the NCCT scan to provide relevant information when surgical intervention is required.

A comprehensive, conclusive clinical approach to identify patients with a high likelihood of ureterolithiasis, low risk of alternative diagnosis and a low risk of surgical intervention remains to be formulated. This clinical approach is likely to rely on clinical, laboratory and US findings.

## Figures and Tables

**Table 1:** Baseline characteristics of entire cohort of patients (SD = standard deviation; IQR = interquartile range).

	Baseline Characteristics, *n* = 368
Age, mean, years (SD)	47.2 (13.9)
Gender	
Male, %	84.2
Female, %	15.8
Duration of symptoms, mean, days	4.1
Visual Analog Scale score, median (SD)	8 (3.4)
Nausea and vomiting, %	59.7
History of urolithiasis, %	42.4
History of intervention for urolithiasis, %	8.7
Serum creatinine level, mean, mg/dL (SD)	1.2 (0.4)
WBC count, mean, k/μL (SD)	11.2 (3.3)
Stone Location	
Proximal, %	29.9
Distal, %	70.1
Right, %	44
Left, %	56
Stone size, mean, mm (SD)	5.0 (2.4)
Admission, %	48.4
Readmission, %	13.3
Intervention, %	36.1
Median time to intervention, days (IQR)	2 (1–5)

**Table 2:** Baseline characteristics of patients who required surgical intervention (Group 1) vs. patients who were successfully treated conservatively (Group 2). Boldface indicates statistically significant factors.

	Group 1 *n* = 133	Group 2 *n* = 235	*p*-Value
Age, mean, years	50	46	***0.003***
Gender			
Male	83%	85%	*0.544*
Female	17%	15%	
Duration of symptoms, mean, days	6.5	2.7	***<0.002***
Visual Analog Scale score, median	8	8	*0.224*
Nausea and vomiting	57%	61%	*0.518*
History of urolithiasis	53%	36%	***0.002***
History of intervention for urolithiasis	13%	6%	***0.033***
Serum creatinine level, mean, mg/dL	1.32	1.14	***0.001***
WBC count, mean, k/μL	10.9	11.3	*0.295*

**Table 3:** Multivariate analysis of predictors for surgical intervention. Boldface indicates statistically significant factors.

	OR (95% CI)	*p*-Value
**Creatinine > 1.5 mg/dL**	**2.8 (1.5–5.6)**	***0.002***
**Duration of Symptoms > 1.5 days**	**2.3 (1.4–3.8)**	***0.001***
**Age > 45 years**	**1.7 (1.02–2.7)**	***0.042***
History of urolithiasis	1.4 (0.8–2.4)	*0.186*
History of intervention for urolithiasis	2.0 (0.8–5.1)	*0.118*

**Table 4:** Stone location and size according to age, creatinine level and duration of symptoms.

Clinical Factor	Stone Size mm (SD)	Proximal	Distal
Age < 45 years	4.6 (1.9)	24%	76%
Age > 45 years	5.4 (2.6)	34%	66%
	***p = 0.001***	***p = 0.039***	
Cr < 1.5 mg/dL	4.8 (2.0)	27%	73%
Cr > 1.5 mg/dL	6.4 (3.7)	47%	53%
	***p = 0.002***	***p < 0.002***	
Symptoms < 1.5 days	4.7 (2.4)	28%	72%
Symptoms > 1.5 days	5.5 (2.4)	36%	64%
	***p = 0.002***	***p = 0.047***	
No Clinical Factors	4.2	22%	78%
All Clinical Factors	6.7	54%	46%
	***p < 0.002***	***p = 0.002***	

## Data Availability

The data presented in this study are available on request from the corresponding author. The data are not publicly available due to health fund and hospital policy regarding patient confidentiality.
